# Histology segmentation using active learning on regions of interest in oral cavity squamous cell carcinoma

**DOI:** 10.1016/j.jpi.2022.100146

**Published:** 2022-09-27

**Authors:** Jonathan Folmsbee, Lei Zhang, Xulei Lu, Jawaria Rahman, John Gentry, Brendan Conn, Marilena Vered, Paromita Roy, Ruta Gupta, Diana Lin, Shabnam Samankan, Pooja Dhorajiva, Anu Peter, Minhua Wang, Anna Israel, Margaret Brandwein-Weber, Scott Doyle

**Affiliations:** a Department of Pathology & Anatomical Sciences, University at Buffalo SUNY, Buffalo, NY, USA; b Department of Biomedical Engineering, University at Buffalo SUNY, Buffalo, NY, USA; c Icahn School of Medicine, The Mount Sinai Hospital, New York, NY, USA; d Department of Pathology, Case Western University, Cleveland, OH, USA; e Department of Pathology, Nebraska Medical Health System, Omaha, NE, USA; f Department of Pathology, University of Edinburgh, Edinburgh, UK; g Department of Oral Pathology, Oral Medicine and Maxillofacial Imaging, School of Dental Medicine, Tel Aviv University, Tel Aviv, IL, USA; h Institute of Pathology, Sheba Medical Center, Tel Hashomer, Ramat Gan, IL, USA; i Department of Pathology, Tata Memorial Cancer Center, Mumbai, IN, USA; j Department of Tissue Pathology and Diagnostic Oncology, NSW Health Pathology, Royal Prince Alfred Hospital and University of Sydney, Sydney, AU, USA; k Department of Pathology, The University of Alabama at Birmingham, Birmingham, AL, USA; l Department of Pathology, George Washington University Hospital, Washington, DC, USA; m Department of Oncologic Surgical Pathology, Memorial Sloan Kettering Cancer Center, New York, NY, USA; n Department of Pathology, University of Pennsylvania, Philadelphia, PA, USA; o Department of Pathology, Yale University School of Medicine, New Haven, CT, USA; p Department of Anatomic Pathology, Robert J. Tomsich Pathology and Laboratory Medicine Institute, Cleveland Clinic, Cleveland, OH, USA

**Keywords:** Active learning, Oral cavity cancer, Computational pathology, U-net, Digital pathology, Semantic segmentation, Whole slide imaging, Region of interest

## Abstract

In digital pathology, deep learning has been shown to have a wide range of applications, from cancer grading to segmenting structures like glomeruli. One of the main hurdles for digital pathology to be truly effective is the size of the dataset needed for generalization to address the spectrum of possible morphologies. Small datasets limit classifiers’ ability to generalize. Yet, when we move to larger datasets of whole slide images (WSIs) of tissue, these datasets may cause network bottlenecks as each WSI at its original magnification can be upwards of 100 000 by 100 000 pixels, and over a gigabyte in file size. Compounding this problem, high quality pathologist annotations are difficult to obtain, as the volume of necessary annotations to create a classifier that can generalize would be extremely costly in terms of pathologist-hours. In this work, we use Active Learning (AL), a process for iterative interactive training, to create a modified U-net classifier on the region of interest (ROI) scale. We then compare this to Random Learning (RL), where images for addition to the dataset for retraining are randomly selected. Our hypothesis is that AL shows benefits for generating segmentation results versus randomly selecting images to annotate. We show that after 3 iterations, that AL, with an average Dice coefficient of 0.461, outperforms RL, with an average Dice Coefficient of 0.375, by 0.086.

## Background and motivation of the work

### Labeled training data in computational pathology

Deep learning (DL) can achieve state-of-the-art performance on a wide variety of computer vision tasks related to computational pathology.^1^, ^2^, ^3^

**Fig. 1:**
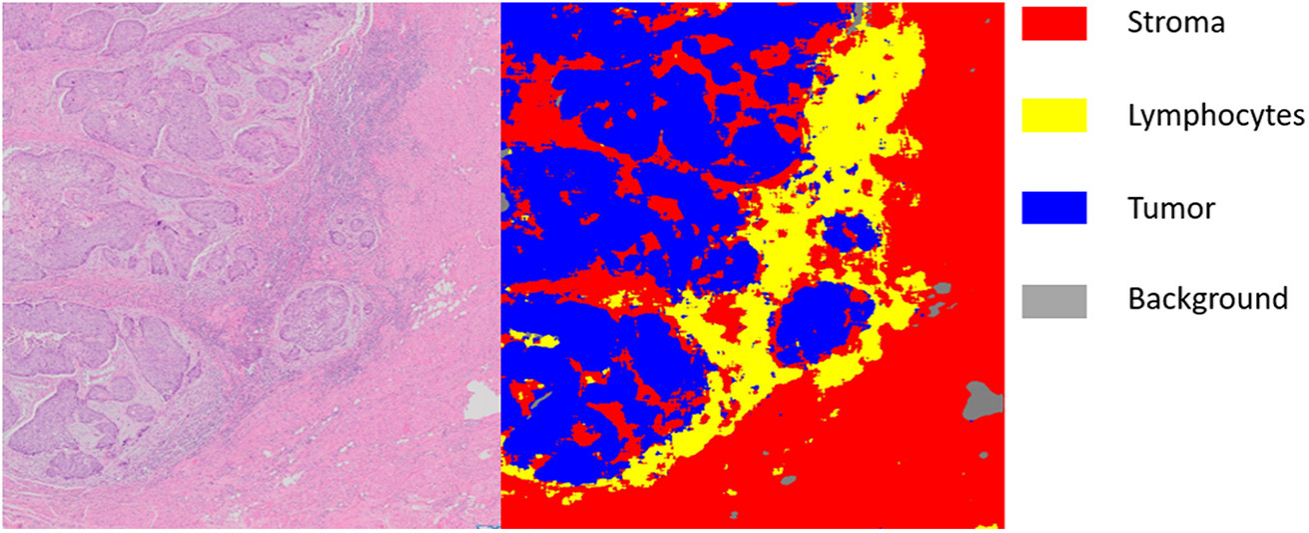
An example of semantic segmentation on oral cavity cancer.

One of the most challenging areas of computational pathology is the multi-class segmentation of brightfield hematoxylin and eosin (H&E)-stained tissue images, where each pixel in the image is assigned to a class, as shown in Fig. 1. For cancer, the list of segmentation classes may include tumor, lymphocytic response, and normal stroma or epithelial tissue, all of which may indicate the aggressiveness of the tumor and likely treatment or outcome predictions. The results of segmentation can then be leveraged for quantifying tumor growth patterns, like lymphovascular and perineural invasion, or measuring important morphological or architectural features.^4^, ^5^, ^6^

DL algorithms require a large amount of labeled training data to be successful. The total number of samples for a given problem are difficult to estimate *a priori*, but are dependent on the complexity and variation of class appearances, number of classes, and the size of the input data.^7^

As these factors increase, the dataset size must grow accordingly. Furthermore, to prevent overfitting and demonstrate generalizability of a controlled DL experimental setup, the fully annotated dataset of images must be divided into disjoint training, validation, and testing groups, which further increases the total number of required labeled samples.^8^^,^^9^

It is challenging to obtain a large and comprehensively labeled dataset, both at the data level (i.e., whole-slide scanning) and at the annotation level. While “natural” image datasets are amenable to crowd-sourced generation and annotation,^10^ pathological images require highly specific training to accurately annotate, as in Fig. 1. Annotating data for segmentation involves pixel-level delineation of multiple classes, which is time-consuming and difficult, particularly as some pathological classes of interest (e.g., lymphocytic host response) do not have precisely defined spatial boundaries. Variation among annotators is common, particularly for classes with confounders or those that are difficult to precisely identify on a digital whole slide image. The “type” of annotation (bounding box, pixel-level, etc.) can also vary, leading to differences in the amount of time required for generating labeled datasets. Due to these challenges, publicly available datasets for pathology segmentation are task-specific, focusing on cellular structures,^11^ architectural structures,^12^ or tissue compartments.^13^ This means that the work of generating new segmentation datasets is time-consuming, expensive, and must be done from the ground up for each pathological process.

### Strategies to circumvent annotation burden

These challenges have been addressed by recent advances in DL training for computational pathology. These advances include transfer learning, zero- and one-shot training, and unsupervised and semi-supervised approaches.

Transfer learning is the process of using a previously trained DL model to “jump-start” the training of a new model. In this approach, the new model is initialized with a parameter set from a model with similar architecture which has been trained on a large, well-annotated dataset.^14^ After initialization, the model is “fine-tuned” to recognize the specific classes in the target dataset. The intuition behind this approach is that tasks in a given domain like computer vision require similar content descriptors; these descriptors are defined by the weights associated with the layers of the network architecture. By jump-starting the system with a set of pre-trained parameters, a new domain-specific dataset will require fewer rounds of training and less annotated data.

Transfer learning is a powerful tool for reducing training set sizes, but is highly dependent on the similarity between the “source” (initial) and the target dataset. Training a network to recognize natural images does not necessarily prepare it to do well at classifying H&E stained microscopy.

**Fig. 2:**
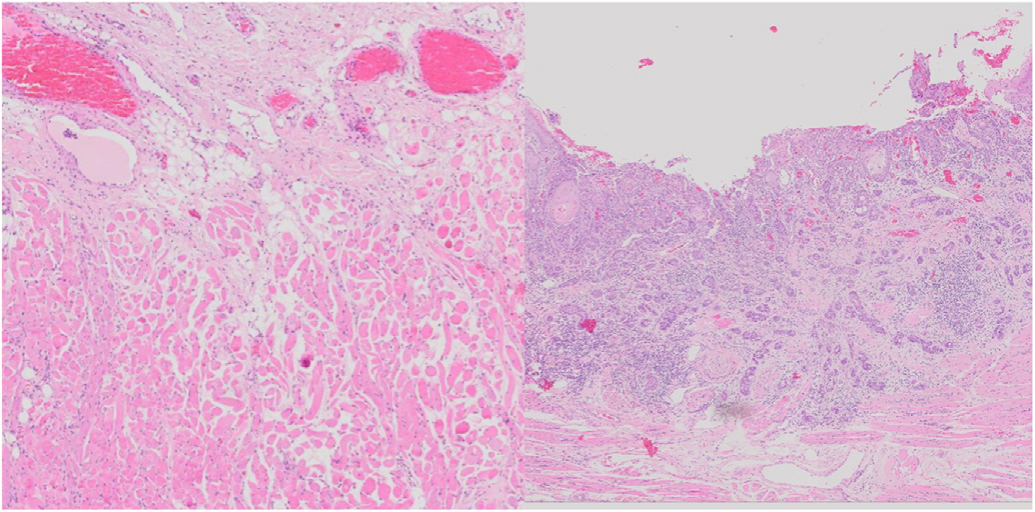
An example of a less informative sample (left) vs a more informative sample (right) in looking for worst pattern of invasion. The image on the left has very sparse lymphocytic infiltration and little tumor, whereas the image on the right showcases tumor and tumor satellites as well as more distinct and dense regions of lymphocytic infiltration.

Zero- and one-shot training are methods that attempt to identify outliers prior to model training, so that “informative” samples can be identified *a priori* and annotated.^15^^,^^16^ An example of the informative differences in samples is shown below in Fig. 2

The challenge with this training approach lies in the definition of “informative” samples: often, the variability among a single class pattern is so great that it is difficult to identify outliers relative to the baseline class structure. To properly identify informative samples, an expert pathologist would be required to review and label samples according to the likelihood that they will improve classification performance.

The abundance of unlabeled H&E-stained cancer datasets have given rise to unsupervised or semi-supervised training methods, where labeled samples are either not used or are a minority of the total training set.^17^, ^18^, ^19^ In these methods, cluster relationships are used as a primary source of information about class membership, relying on the latent data structure (as defined by image content descriptors) to distinguish semantically meaningful areas of the image, where there are many classes with differing colors and intensities.

While these methods can help to bootstrap a segmentation approach between classes with distinct color and intensity contrast, they are not suitable for highly variable image patterns, confounders, or a large number of classes, as we might expect to find in a whole-slide tissue sample.

Therefore, unsupervised models are insufficient for acquiring usable results for complex tasks such as tissue labeling, and fully supervised labels are preferable to train semantic segmentation models.

#### Active and human-in-the-loop learning

Active learning (AL)^20^^,^^21^ is a supervised, iterative training method that combines aspects of semi-supervised and one-shot learning. In AL, a bootstrap model trained on a small subset of annotated data is allowed to classify a set of unlabeled, “potential” training data. Based on some criteria, these classified samples may be selected for manual re-labeling, after which they are added to the bootstrap training set. There are several ways to define this selection criteria based on the type of information the designer seeks to maximize. Unsupervised clustering-based methods are designed to use the structure of the feature space to highlight samples of interest, ensuring that informative samples (those that represent potentially new classes, or outlier samples) are preferentially added to the dataset.^22^ Other approaches focus on sample “uncertainty”, quantified directly by probabilistic classifiers or estimated for samples based on difficulty of classification (e.g., closeness to a boundary in Support Vector Machine-based methods, or disagreement among committee-based approaches).^23^^,^^24^ All of these approaches seek to reduce the number of training samples that require manual annotation. The hypothesis of AL is that by iteratively introducing new samples that maximize classifier performance rather than randomly selecting and annotating new samples, the performance of the resulting classifier will be higher with a small number of samples (or, similarly, that the classifier will reach a “target” level of performance with fewer training samples). AL does not necessarily improve *final* classifier performance, but instead seeks to reach that final performance with fewer samples compared to random learning (RL).

A closely related concept to AL is human-in-the-loop (HITL) training. In this approach, a human is tasked with manually reviewing and adjusting the training data or design of an AI system. HITL systems can be used to make sure the results of AI are accurate, explainable, and in line with the intended application.^25^, ^26^, ^27^ Previous groups have used HITL to great effect for whole-slide digital pathology segmentation. Lutnick et al. used a system (termed HAI-L, for “Human-AI-Loop”) to iteratively improve segmentation of glomeruli structures in kidney biopsies^28^ and found that the time of annotation required for classifier performance was greatly reduced.

In this paper, we combine these training approaches, using manual assessment of classifier performance as the criteria for selecting new samples for full re-annotation and inclusion into the training set. The hypothesis of this work is that HITL and AL will enable human control of the AI tuning (identifying mislabeled samples as well as new classes to add to training), and that the classification performance will improve faster with AL when compared with RL.

### Application: Oral cavity cancer overview

In this work, we apply our segmentation training approach to a dataset of H&E stained Oral Cavity Cancer (OCC) tumor whole slide images (WSIs). In 2021, OCC was newly diagnosed in 53 260 patients and resulted in 10 750 deaths in the United States, with 377 713 cases being diagnosed worldwide in 2020. Overall, the disease has a 5 year predicted survival rate of 57%.^29^^,^^30^ The staging system for OCC is divided into low (Stage I/II) and high (Stages III/IV) stage. Low-stage patients are typically treated with surgery alone, whereas high-stage patients receive adjuvant chemoradiotherapy. Unfortunately, 25% of Stage I patients and 37% of Stage II patients will experience loco-regional recurrence (LRR). The Histologic Risk Model (HRM)^31^ was developed for OCC using 3 histological variables to identify high-risk patients: Worst Pattern of Invasion (WPOI), Lymphocytic Host Response (LHR), and Perineural Invasion (PNI). Of these 3, WPOI was found to be the most significant variable with the greatest predictive performance. The HRM has been clinically validated,^32^, ^33^, ^34^, ^35^, ^36^, ^37^, ^38^, ^39^ but it has not seen broad use in the clinic due to the difficulty of translating the criteria into pathological practice.

Our overarching goal is to develop a computational Quantitative Risk Model (QRM), based on known priors of the HRM. A laboratory-developed digital QRM test can theoretically refine and improve upon the HRM, enhance its robustness, and increase the availability of risk scoring. In this work, we aim to provide segmentation of WSIs on tumor resected images, creating “tumor maps”. Features can be extracted from these tumor maps for risk-stratification. We evaluate our combined human-in-the-loop and AL training pipeline to build a multi-class semantic segmentation classifier which can identify structures of interest relevant to the HRM.

## Methods

### Image dataset creation

The overall dataset consists of 151 whole slide images (WSIs) from 107 clinically low stage OCC patients which were consecutively accrued.

Tumor resection slides generated during normal course of treatment were stained with Hematoxylin and Eosin (H&E) and digitized via an Olympus scanner at 0.167 microns per pixel, or 40x magnification. Only the most informative slides were digitized, at the discretion of the pathologist. Specimens were blocked in their entirely, and were processed via standard hospital clinical procedures in the histopathology field. Whole slides were selected via the criteria of WPOI, PNI, and LHR from the histological risk model. Regions of interest from the whole slides were selected manually via the criteria of WPOI, as the time pathologists had to generate labels was constrained, and as previously mentioned WPOI has the greatest predictive performance.

As a result, multiple WSIs can come from the same patient, depending on the size and complexity of the original tumor resection. Following digitization and de-identification, all WSIs were placed into a Digital Slide Archive database for access and analysis. Pathologists performing WSI selection, ROI selection, and annotating images, were all attending pathologists specifically trained in the field of head and neck cancer, with the cohort coming from 8 different universities across the world.

From the 107 patients, 23 were randomly selected for manual ground-truth annotation. ROIs were selected and cropped by a pathologist from each WSI based on tissue WPOI via the HRM, and these ROIs were hand-annotated. In total, 24 ground-truth maps were created (1 of the 23 patients generated 2 ROI annotations). These label maps were created in Photoshop by pathologists trained in using the HRM.

Each tissue class was assigned a color, and pathologists were instructed to label only classes in which they were highly confident, leaving the remainder of the image as an “avoid” class. A total of 12 tissue classes, listed in Table 1 were identified, not including the “avoid” class. The class called lymphocytes is shortened from lymphocytic host response, and it represents areas of stroma rich in lymphocytes.

**Table 1:** Legend of classes and their colors.

Class name	Annotation color
Stroma	Red
Tumor	Blue
Lymphocytes	Yellow
Mucosa	Sky blue
Background/Adipose	Gray
Blood	Green
Nerves	Orange
Necrosis	Black
Keratin Pearl	Dark blue
Muscle	Olive
“Junk” (tissue folds, out of focus areas, ink)	Pink

During annotation, a pair of classes was identified that have similar presentation in the H&E ROI images. Slide background and adipose tissue both present as light gray/white areas, which are highly contrasting with the surrounding tissue and do not appear like any other class in the tissue list. Because of the difficulty in distinguishing this pair of classes, we have merged them into a “super-class” and labeled them together in the segmentation experiments. Following this fusion, 11 tissue classes remained.

Following annotation, this dataset was further divided into a training dataset (20 patients) and a hold-out testing dataset (3 patients) for use in training and quantitatively evaluating the segmentation algorithm. This split was performed at a patient level, meaning that all ROIs belonging to a patient were placed into the corresponding group (i.e., no patients’ slides appear in both training and testing datasets). Prior to training and evaluation, each annotated ROI was resized to 2 microns per pixel and cropped to pixel dimensions of 2000 × 2000. Image standardization to the calculated mean of the dataset was performed as a preprocessing step.

### Segmentation classifier architecture

**Fig. 3:**
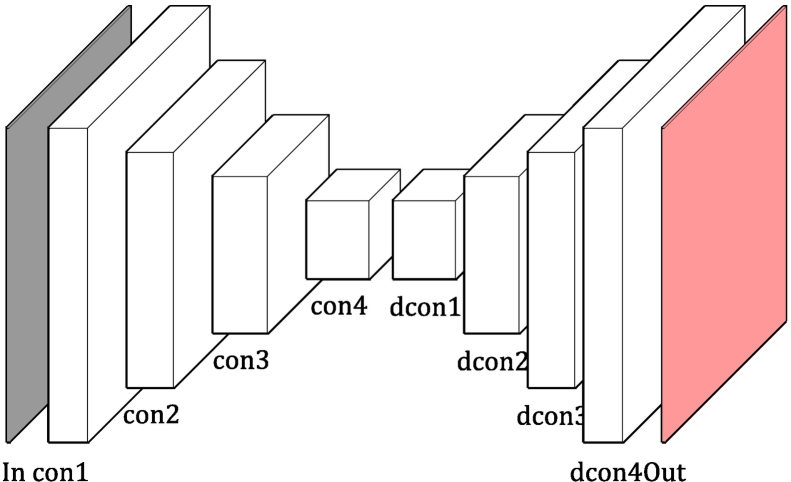
Visual representation of utilized CNN architecture.

The segmentation classifier is a simple modification of the U-net architecture.^40^ Our version is shown in Fig. 3, consisting of a set of 4 down-sampling convolutional blocks connected to a symmetrical set of 4 up-sampling deconvolutional blocks.

Each down-sampling block consists of a space-preserving 2D convolutional layer (kernel size=3), batch normalization, and ReLU nonlinear layers, followed by 2 × 2 maximum pooling layers to reduce the size of the input by half. Each up-sampling block consists of an up-sampling layer followed by a space-preserving 2D convolutional layer, batch normalization, and ReLU nonlinear layer. At each up-sampling block, the outputs from the corresponding down-sampling block are concatenated, following the procedure in Ronneberger et al.^40^

### Classifier training pipelines

In our experimental setup, several classifiers were trained and evaluated as described in the sections below. Each classifier was trained for 300 epochs with a learning rate of 1 × 10^−4^, a batch size of 1, and a dropout rate of 0.8 applied after each max pooling layer.

#### Active learning training approach

**Fig. 4:**
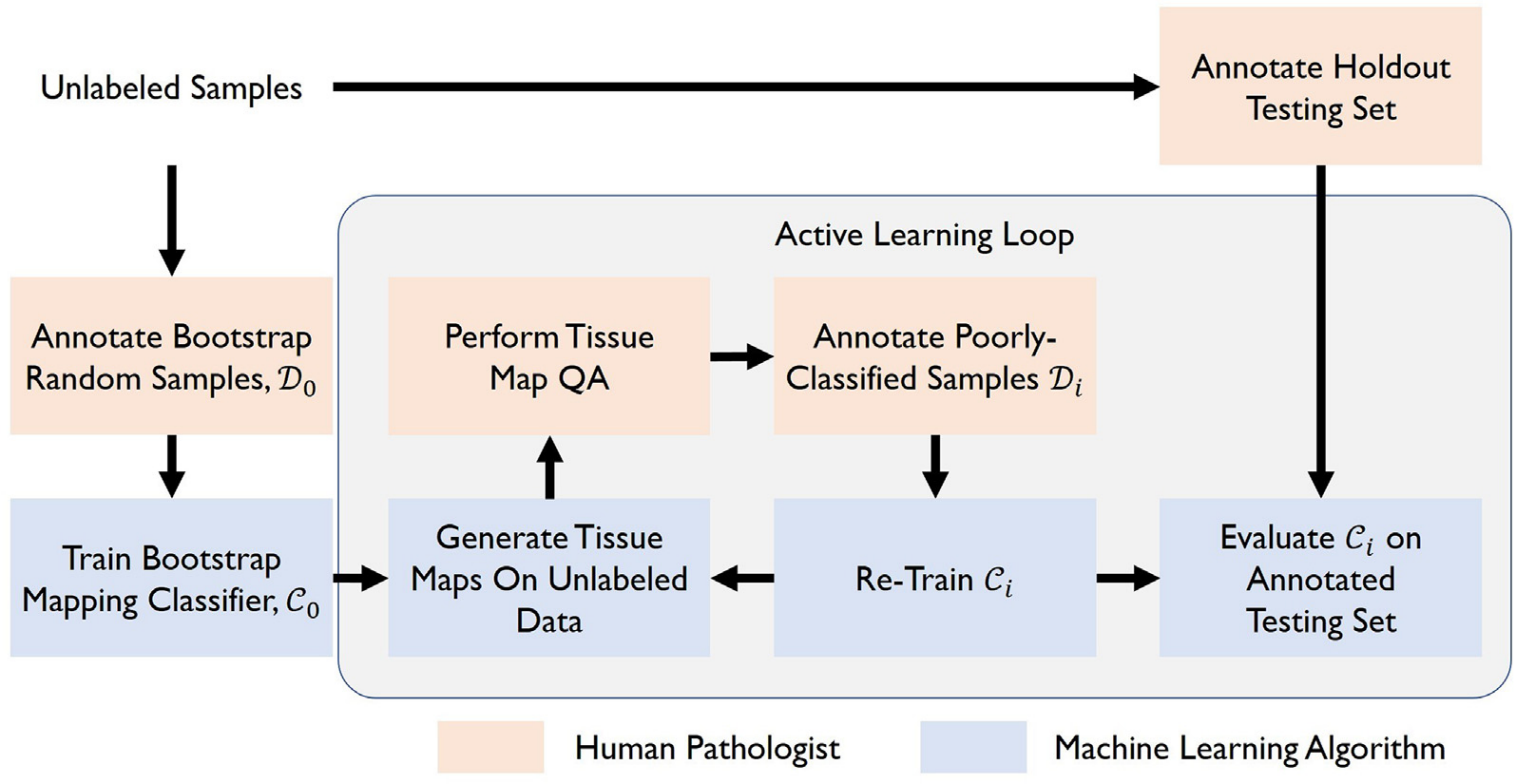
Active learning pipeline emphazing the roles of the AI and the pathologists.

Our human-in-the-loop AL pipeline is summarized in Fig. 4. This is an iterative pipeline, where sets of training samples $D_{i}$ are used to train classifiers $C_{i}$, where *i* represents the training iteration.

We begin with the pool of 24 samples identified for use in training. From this set, 3 of these samples were removed and used as an independent holdout testing set, leaving 21 samples for potential inclusion in classifier training.

From this pool, a small subsample of 4 ROIs was randomly selected and added to the first training dataset iteration, denoted $D_{0}$. These samples were used to train a “bootstrap” classifier, denoted $C_{0}$. The remaining 17 samples in the training pool were then segmented by $C_{0}$ to yield a set of tissue maps.

Training then proceeded iteratively. At each iteration *i*, the tissue maps generated by $C_{i}$ (along with the image ROIs themselves) were analyzed in a “Tissue Map QA” process, where each image was graded qualitatively by a team of pathologists on a scale of 0–5 for each tissue class. A score of 5 represented an ideal or “perfect” segmentation and 0 represented a poor segmentation. The 4 images with the lowest scores were then added to the training set to create a new AL training set, $D_{i+1}$, and the classifier was re-trained to yield $C_{i+1}$. This was done iteratively until *i* = 3. At each iteration, classifier $C_{i}$ was evaluated quantitatively on the holdout testing examples as described below.

#### Active learning metrics and evaluation

At each training iteration, the classifier $C_{i}$ was evaluated using 3 metrics: Categorical Cross-Entropy Loss, Sørensen-Dice coefficient, and Receiver Operating Characteristic (ROC) curve analysis.

To calculate the loss, we used the categorical cross entropy loss function, which is calculated as:

$$L\left(x,y\right)=-\log\frac{\exp\left(x_{j}\right)}{\sum_{c=1}^{C}\exp\left(x_{c}\right)}$$

where the *x*_*j*_ represents samples for which the predicted class does not match the annotated class (i.e., mistakes) and *C* is the total number of classes in the classifier output.

Sørensen-Dice coefficients were calculated as:

$$\mathrm{dice}\left(c\right)=\frac{2^{*}{\mathrm{TP}}_{c}}{\left(2^{*}{\mathrm{TP}}_{c}+{\mathrm{FP}}_{c}+{\mathrm{FN}}_{c}\right)}$$

where *TP*_*c*_, *FP*_*c*_, and *FN*_*c*_ represent the true-positive pixels, false-positive pixels, and false-negative pixels, respectively, for tissue class *c*. These values were calculated across the holdout testing set to yield a set of Dice coefficients for each class.

Similarly, ROC curves were calculated on a class-by-class basis using a “one vs all” strategy. For each class, the area under the ROC curve (AUC) was calculated as an overall measure of performance balancing sensitivity and specificity. Finally, as our initial holdout testing set was small, 32 additional ROIs from 31 patients not present in the training set were extracted post iteration through the AL pipeline to augment the holdout testing set. We also performed qualitative evaluation of the resulting computer generated label maps compared to the ground truth.

#### Random learning training approach

The control set of our experiments is a random learning (RL) training paradigm. In this scenario, training set $D_{0}$ is the same as in AL. At each iteration *i* of training, we added a random set of 4 ROIs to create ${\hat{D}}_{i+1}$, which in turn was used to generate classifier ${\hat{C}}_{i+1}$, where $\hat{D}$ and $\hat{C}$ represent randomly selected training sets and classifiers, respectively. In addition, RL training was performed 3 times to yield multiple random batches of $\hat{D}$.

#### Random learning metrics and evaluation

Each classifier ${\hat{C}}_{i}$ was evaluated using the same quantitative metrics as described above for AL. We used the mean of the 3 RL training runs to compare with the single AL run. In addition, we also recorded the standard deviation of the performance metrics to see how variable RL training pipeline is with randomly selected samples.

## Results

### Training and validation loss

**Fig. 5:**
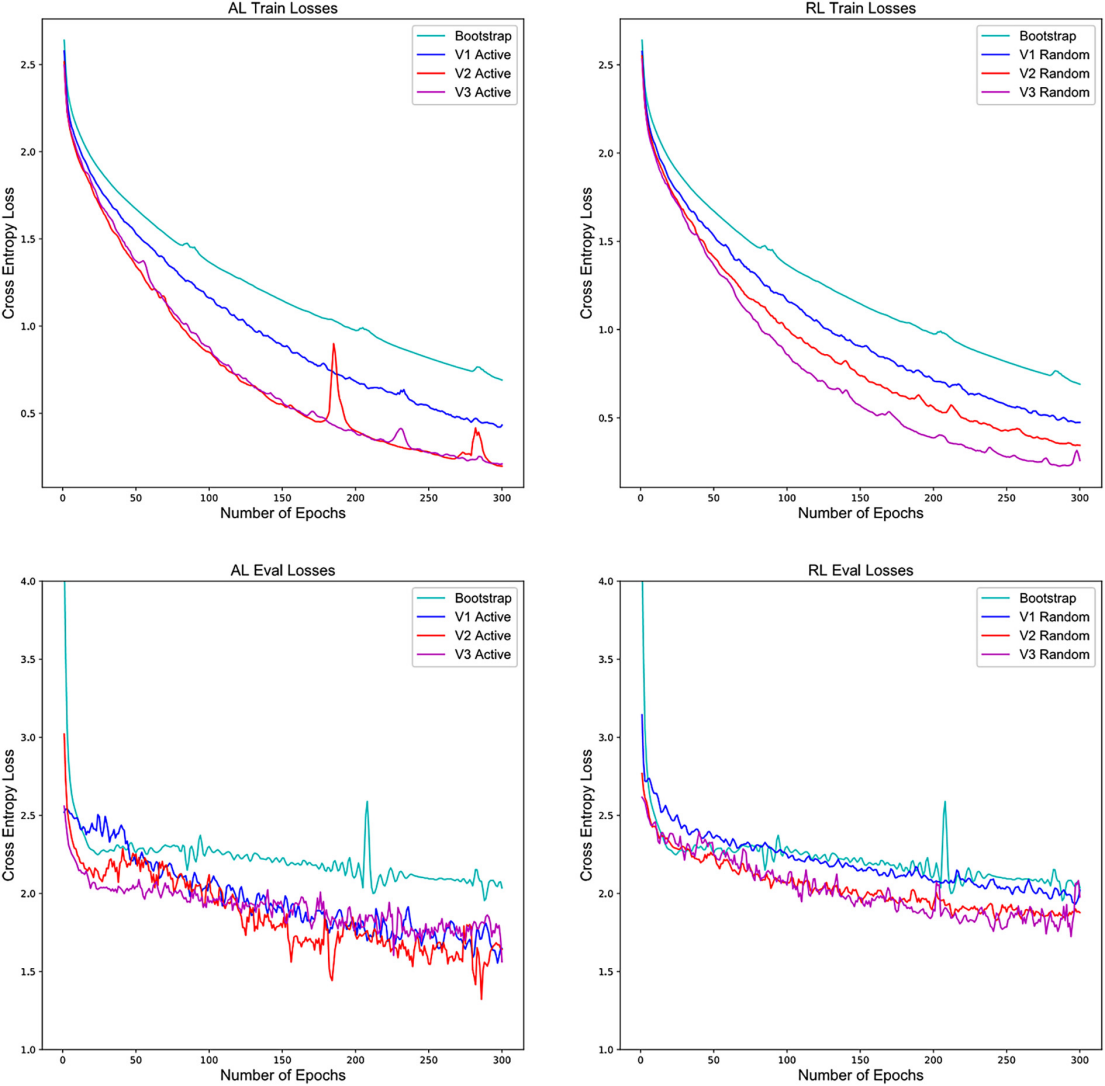
Loss curves for training and validation across different iterations of AL and RL. While the training curves are similar, we see validation losses for AL are lower across versions than RL.

After 4 iterations, it was found that while there is no significant difference in training loss between versions of AL and RL, the validation loss for the AL was lower than the mean of the RL for every version. The loss plots for training and evaluation can be seen in Fig. 5. Loss for each of these was calculated as a 3 epoch average for each point on the graph, and the loss curves shown for RL are the average of all 3 batches.

### Quantitative testing set performance

#### Classification performance

**Fig. 6:**
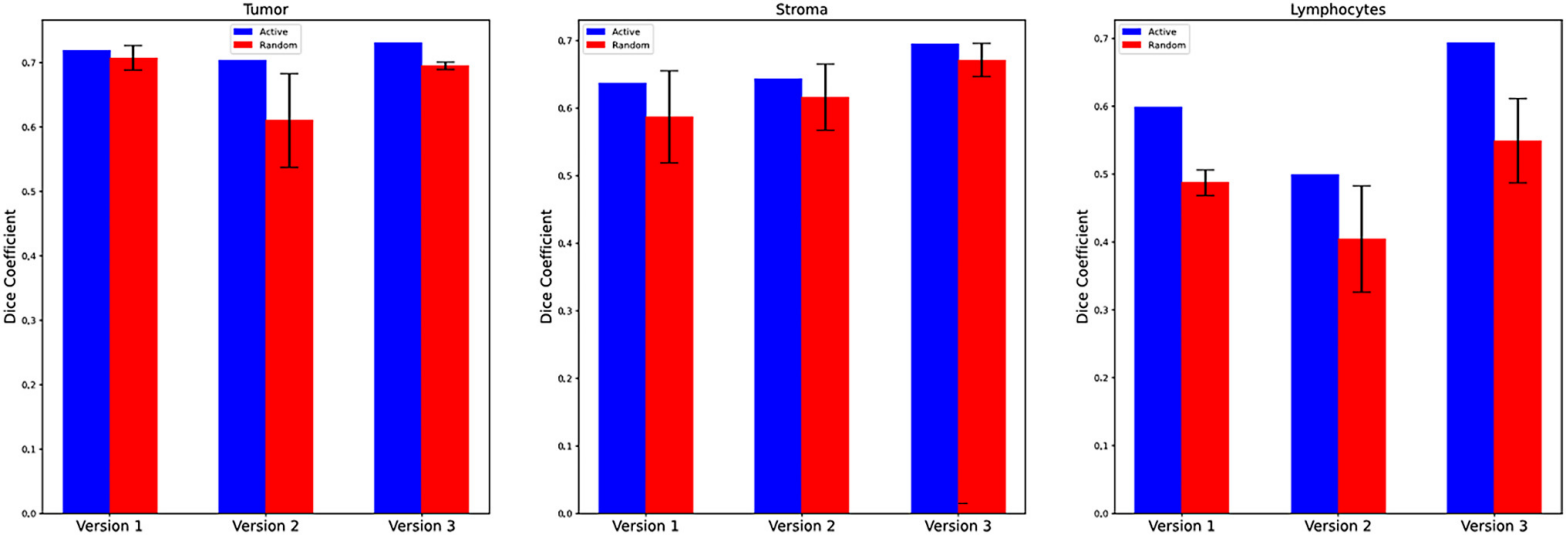
Dice coefficients across all versions for tumor, stroma, and lymphocytes. This demonstrates the varying degrees of impact AL has across different classes of interest.

**Fig. 7:**
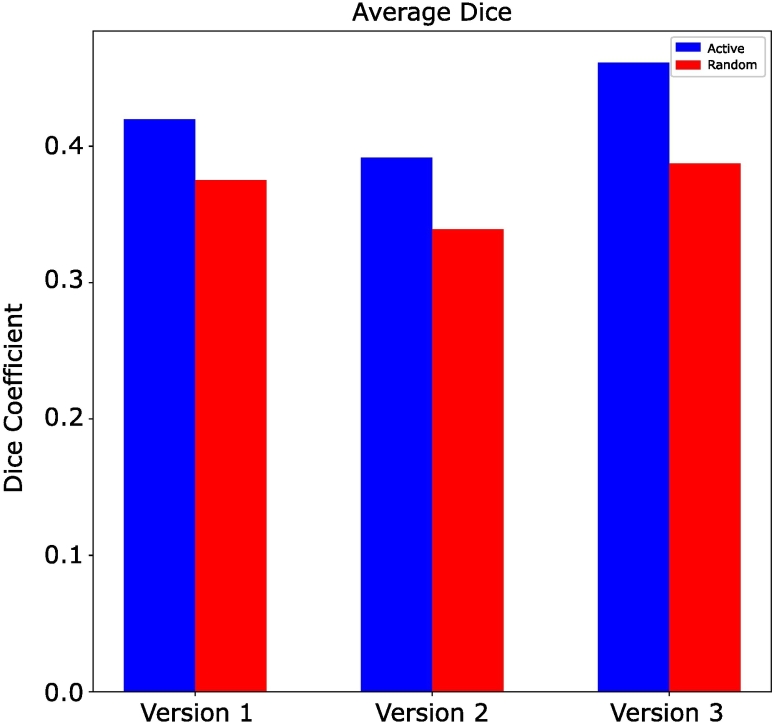
Unweighted average dice coefficient across all versions of AL vs RL(p=0.011).

The Dice coefficients for all present classes in the holdout testing images are shown in Table 2. Dice for the RL versions are averages of the 3 batches. Since AL only had 1 training vs the 3 separate batches of RL, there is no variation for AL. The Dice coefficients in version 3 of the AL were all higher for the classes present in the holdout testing images than the Dice coefficients in version 3 of the RL. The average of all Dice coefficients are shown up in Fig. 7 and are broken down for the tumor, stroma, and lymphocyte classes in Fig. 6.

**Table 2:** Dice coefficients for present classes for holdout testing images across all versions. The highest dice coefficient for each class is in bold text.

	1AL	2AL	3AL	1RL	2RL	3RL
Tumor	0.719	0.703	**0.723**	0.708	0.610	0.695
Stroma	0.636	0.643	**0.695**	0.587	0.616	0.671
Lymphocytes	0.599	0.498	**0.692**	0.487	0.404	0.549
Mucosa	0	0.006	0.002	0	0.002	**0.010**
Blood	0	0.004	**0.242**	0.186	0.197	0.207
Keratin pearl	0.077	**0.363**	0.189	0.172	0.164	0.008
Muscle	0.077	0.012	**0.116**	0.011	0.056	0.008
Background/Adipose	**0.627**	0.517	0.564	0.475	0.326	0.507
Average	0.420	0.391	**0.461**	0.375	0.339	0.375

**Fig. 8:**
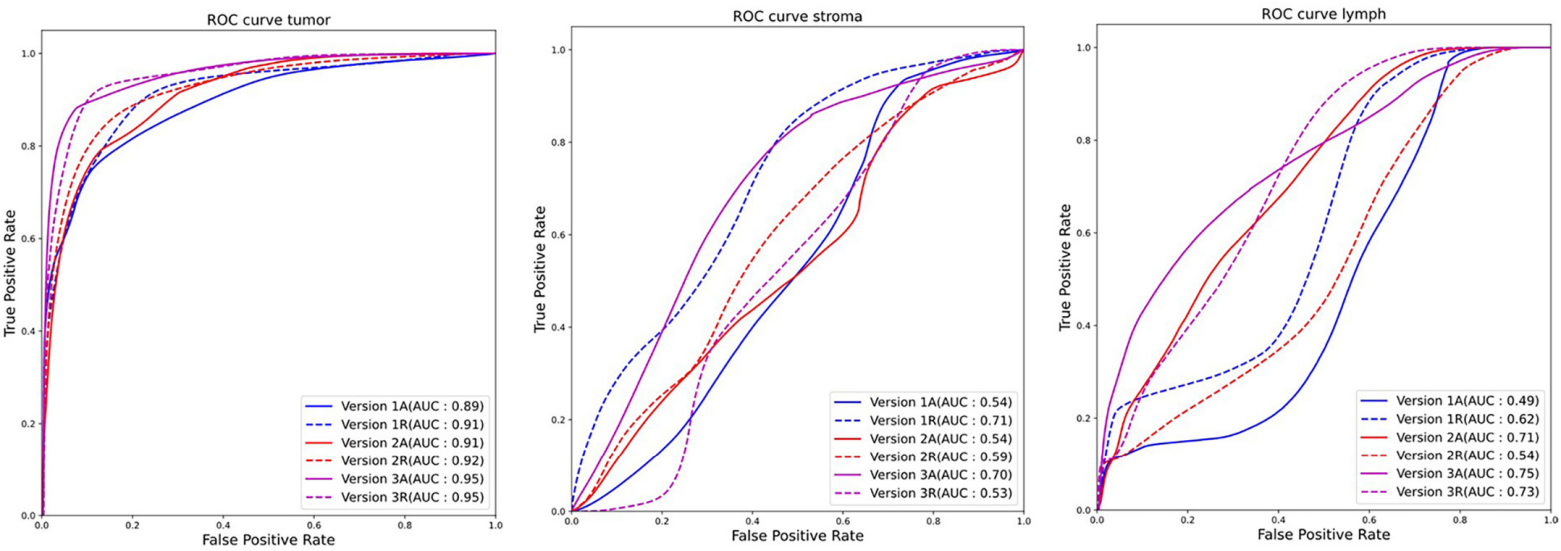
ROC curves for holdout testing images for tumor, lymphocytes, and stroma across all versions.

**Fig. 9:**
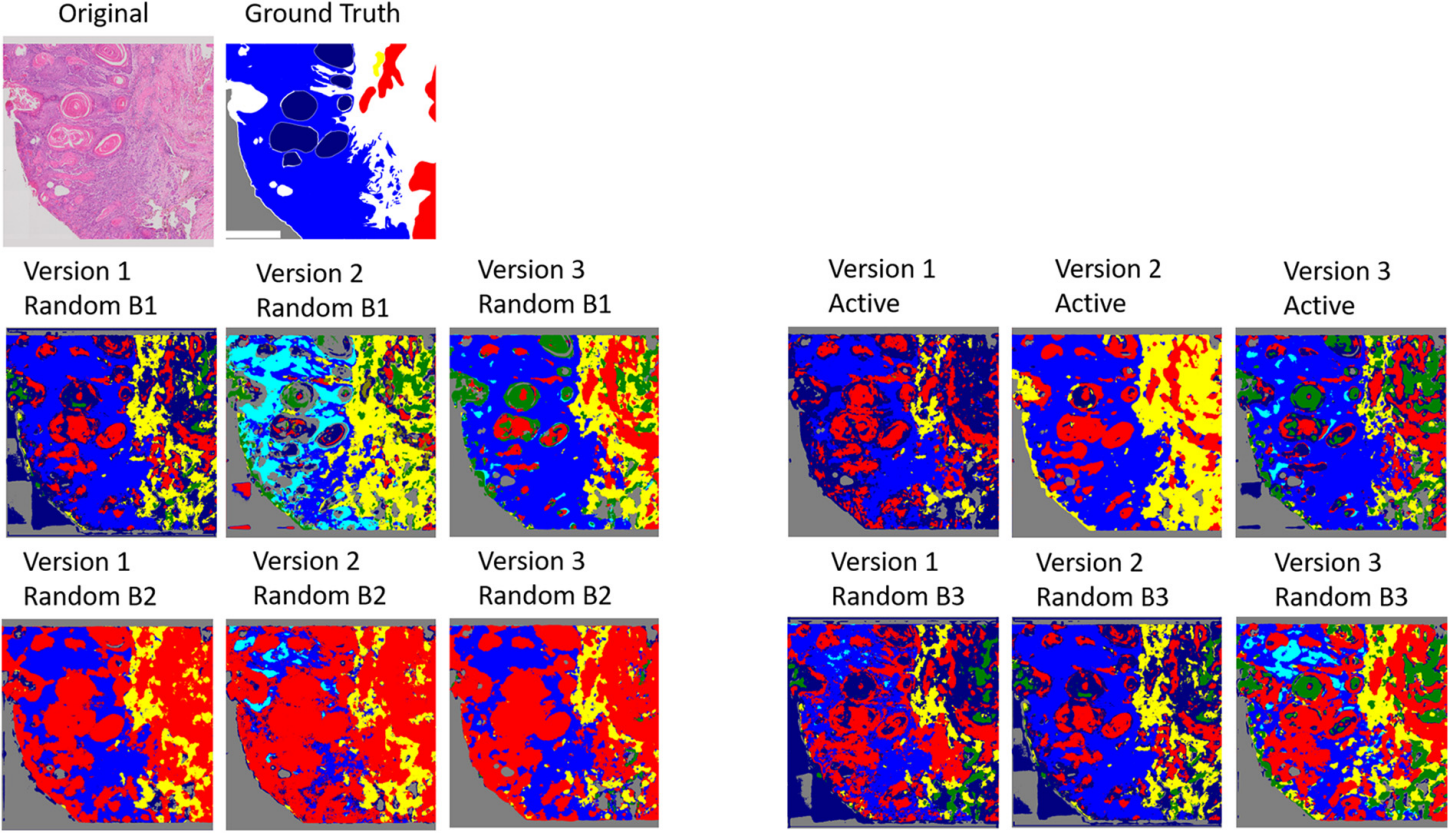
Progression of an ROI for AL vs all 3 batches of RL. We see how RL varies wildly between batches, whereas AL gives a guarantee of qualitative performance.

**Fig. 10:**
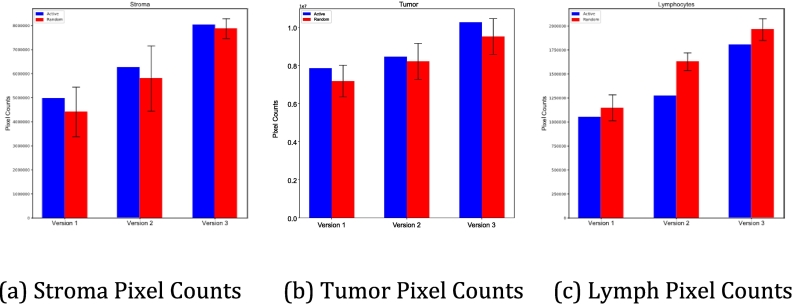
Total ground-truth pixels for classes of interest. This showcases that there is not a statistically significant difference in the amount of pixels of ground truth added via AL vs those added for RL.

**Fig. 11:**
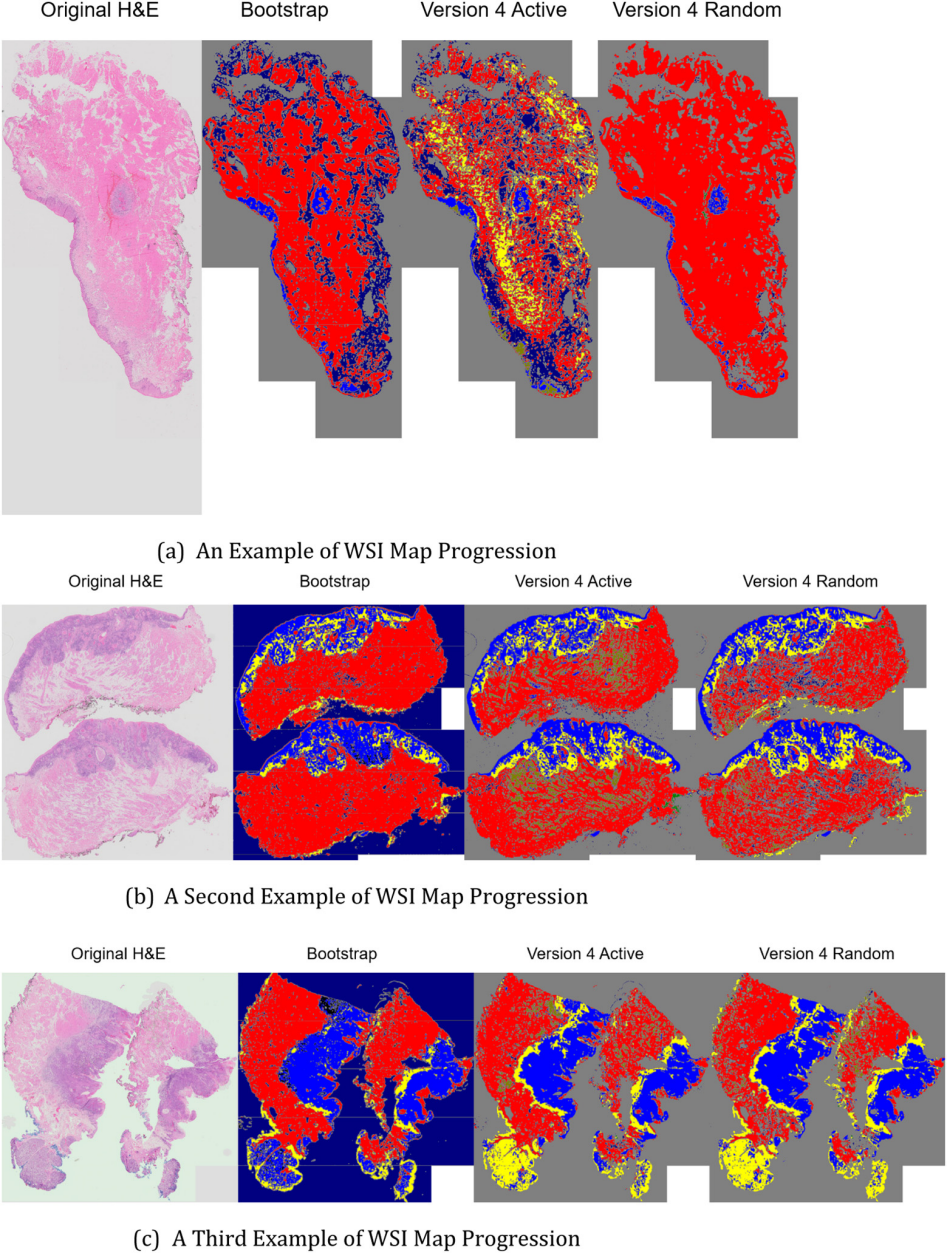
Progression of WSI maps generated for AL vs RL. These demonstrate that the AI WSI maps improve for both AL and RL across versions.

#### Segmentation sensitivity and specificity

Table 3 shows the AUC for all classes across all versions. Fig. 8 shows ROC curves and AUC for the holdout testing set for the most prevalent classes, with the AL and mean RL ROC curves being displayed for each iteration. After statistical testing, we have found no significant difference in AUC for AL vs RL.

**Table 3:** AUC for holdout testing images across all versions. The highest AUCs for each class are in bold text.

	1AL	2AL	3AL	1RL	2RL	3RL
Tumor	0.89	0.91	**0.95**	0.91	0.92	**0.95**
Stroma	0.54	0.54	0.7	**0.71**	0.59	0.53
Lymphocytes	0.49	0.71	**0.75**	0.62	0.54	0.73
Blood	0.49	0.36	0.51	0.71	**0.81**	0.7
Keratin pearl	0.74	0.79	0.79	0.87	**0.92**	0.8
Muscle	**0.61**	0.06	0.36	0.28	0.55	0.17
Background/Adipose	1	1	1	1	1	1

### Qualitative ROI results

Fig. 9 demonstrates the progression of an ROI from Version 1 to Version 3 for AL and the 3 separate batches of RL. The AL ROI maps are more stable across versions than the RL ROI maps.

## Discussion

Active learning shows both qualitative and quantitative benefits on the ROI scale. With a constant dataset size AL outperforms RL. This is shown in the validation losses for AL being consistently lower across versions than RL, and the Dice coefficients performing significantly better for AL vs RL (p=0.011), while the AUCs of the ROC curves maintain their performance. In addition to this, the qualitative AI tissue maps remain more consistent across iterations for AL vs RL.

Fig. 10 illustrates the bar plots for the number of ground-truth pixels of classes in each version. As shown, after 3 iterations there is no significant difference in the number of ground-truth pixels added to the dataset in AL vs RL, meaning we aren’t adding more ground-truth pixels in AL. This means that the increase in performance of AL is driven by how informative the data being added to the dataset is, and not the amount of data. This leads to the conclusion that for any given training set size, AL will outperform RL.

In general, Version 3AL outperformed every other classifier. On a class-by-class basis, as shown by the Dice coefficient and AUC tables, this isn’t as cut and dry. As shown in Table 2, the highest performing classifiers for the keratin pearl and background classes were Versions 2AL and 1AL, respectively. In addition to this, there are also times where more data was added, yet performance decreased, the most notable example of which is for the lymphocyte class showing sharp dips from Versions 1AL and 1RL to Versions 2AL and 2RL. A possible cause of this is the small training set size, and that individual additions to training can have outsized negative effects.

Summing up, first, AL outperforms RL across versions, with Version 3AL performing the best. Even with decreases in performance between different versions and classes, AL outperforms RL for a given dataset size. For us moving to the next step of generating usable WSI AI tissue maps as a starting point for our pathologists, these results make the AL the choice for generating the bootstrap WSI dataset for reannotation.

Examples of what these WSI maps look like when generated are shown in Fig. 11. Shown from left to right in each figure are the original WSI, the Bootstrap result, the AL Version 3 result, and the RL Version 3 result. Qualitatively on the whole slide scale, it shows decent segmentation of tumor and immune host response, however in Subfigure 16 the bottom of the image, which is a gland, is classified as immune host response. This shows the need for reannotation on the WSI scale.

## Concluding remarks and future work

In summary, the ROI AL classifier showed benefits on the ROI scale compared to the RL classifier, with the Dice coefficients for AL outperforming those for RL after 3 versions by an average difference of 0.086, the validation losses being lower for AL than RL, and the AUC curves not being significantly different statistically. This is vital in what we intend on doing in the future, which is human in the loop reannotation for WSIs.

We were able to begin this process by using the ROI classifiers to generate WSI maps as a starting point for our pathologists. Being able to generate segmentation maps on the WSI scale will prove invaluable, as being able to generate a usable starting WSI segmentation for pathologists to work from will reduce annotation burden immensely. The scale of labeled data we are able to add to the dataset in just one pass by giving the pathologists a starting point is orders of magnitude greater than the original ROI annotation pipeline. In addition to this, the cloud server these WSI annotations will sit on will also allow pathologists from different universities to upload their slides and run the newest trained model on them, so we will have more data to validate our model on. One of the other benefits of the cloud server will be that multiple pathologists can reannotate the same image. This will allow us to perform experiments on the variability of annotations, as well as test out different annotation fusion methods.

## Funding source

The funding source for this research is the following RO1 grant obtained by Dr. Scott Doyle:

"A Quantitative Risk Model for Predicting Outcome and Identifying Structural Biomarkers of Treatment Targets in Oral Cancer on a Large Multi-Center Patient Cohort"

National Institutes of Health (NIDCR)

1R01DE028741-01A1

The funding from this grant was used to generated labeled images in the holdout testing set.

## Conflicts of interests

The possible conflicts of interest of those authors listed on the paper are listed below:

Dr. Diana Lin is on the clinical advisory board for Proteocyte AI.

Dr Scott Doyle owns stock options for a digital pathology company called Inspirata, Inc.
